# Frozen-thawed embryo transfer: does the addition of low-dose choriogonadotropin alfa to progesterone in the luteal phase of artificial cycles improve the endometrium and increase the chances of pregnancy?

**DOI:** 10.5935/1518-0557.20230053

**Published:** 2024

**Authors:** Rodopiano de Souza Florêncio, Mylena Naves de Castro Rocha, Vinicius Alves de Oliveira, Marta Curado Carvalho Franco Finotti

**Affiliations:** 1 Humana Medicina Reprodutiva, Goiânia - GO, Brazil

**Keywords:** frozen embryo transfer, luteal phase, choriogonadotropin alfa

## Abstract

**Objective:**

Primary: To evaluate the effect of low doses of recombinant hCG (choriogonadotropin alfa) in the luteal phase of frozen-thawed embryo transfers (FET) of artificial cycles on the chances of pregnancy in patients aged ≤38years. Secondary: To assess the chances of pregnancy in the FET groups of artificial cycles using micronized vaginal progesterone (VP) *versus* injectable intramuscular progesterone (IMP) and the chances of pregnancy in type-1 embryo transfers (two top embryos).

**Methods:**

This retrospective cohort study included 122 cycles of FET and compared two groups of patients aged 38 years or younger, one given hCG in the luteal phase and one not administered hCG.

**Results:**

The clinical pregnancy rates (CPR) in the control and hCG groups were 45% and 45.16%, respectively (*p*=0.9999). The live birth rates (LBR) were 33.33% and 32.25%, respectively, (*p*=0.99909). The CPR in the VP group (83 patients) was 46.89% versus 41.02% in the IMP group, (*p*=0.5459). The LBR was 33.73% in the VP group and 30.76% in the IMP group (39 patients), (*p*=0.7559).

**Conclusions:**

The CPR and LBR of patients undergoing FET in groups prescribed and not prescribed low doses of recombinant hCG were similar. No significant difference was found between patients given VP or IMP.

## INTRODUCTION

The nesting process has been investigated for many years in animals and humans. One of the lines of investigation using fresh embryos looked into human chorionic gonadotropin (hCG) of blastocysts and its role in embryo implantation. Fishel *et al*. (1984) studied the production of hCG in blastocysts from fresh surplus embryos placed experimentally in long culture and found a small amount of ß-hCG with 170 hours of culture and 6033 mIU/ml with 288 hours of culture after in vitro fertilization (IVF). Woodward *et al*. (1993) demonstrated that hCG production by embryos was a time-dependent phenomenon, starting 160 hours after insemination and practically doubling production in less than every 10 hours.

Reshef *et al.* (1990) demonstrated the presence of LH/hCG receptors in the uterus, placenta, fetal membranes, and decidua. In the uterus, the highest expression of these receptors occurred in the endometrium, and in the latter, the highest expression occurred in the cells of the glandular and luminal epithelium, with lower expression in the endometrial stroma.

However, Stewart *et al*. (1999) were unable to document the functionality of these receptors in the endometrium via the analysis of messenger RNA production for LH/hCG receptors in the endometrium, and concluded that these receptors might not be functional. In a literature review, Licht *et al*. (2001) analyzed the role of hCG in the endometrium. The authors described experimental demonstrations of the presence of LH/hCG receptors in the endometrium and the experience of the group with micro intrauterine dialysis associated with intrauterine injections of micro-dose hCG during the luteal phase. They demonstrated that trophoblastic hCG influenced endometrial differentiation, but also observed an evident influence of endometrial products on embryo development, especially leukemia inhibitory factor (LIF).

The intrauterine injection of hCG in microdialysis caused marked effects on the local production of some kinins. The injection increased the production of LIF and decreased the concentration of macrophage colony stimulating factor (M-CSF) and insulin-like growth factor binding protein (IGFBP-1) in the endometrial fluid. They also concluded that hCG had an endocrine effect on the corpus luteum, an autocrine effect on trophoblastic differentiation and a paracrine effect on trophoblastic apposition and invasion.

Perrier d’Hauterive *et al*. (2007) studied the role of a specific receptor for the blastocyst and suggested that LH/hCG-R had an important role in endometrial receptivity. The authors included immunohistochemical analysis of other markers of the implantation window, such as integrin αvβ3, LIF, and interleukin 10 (IL-10), and determined the likely best implantation date through endometrial biopsies.

Golan *et al*. (1993) conducted a prospective randomized study of 60 patients undergoing IVF with an ultrashort agonist regimen and fresh transfers, in which group A received hCG 2500 IU every 3 days after oocyte retrieval and group B was given intramuscular progesterone (IMP) 100 mg daily. They observed statistically higher chances of pregnancy in and high levels of estradiol (E2) and progesterone (PRG) in group A.

Lee *et al*. (2017) evaluated the use of 1500 IU hCG on the day of transfer and 6 days later in natural cycles for frozen embryo transfer (FET) without the use of PRG in the luteal phase. A total of 382 patients were included in the hCG group and 225 in the control group. The percentage of ongoing pregnancy rate (OPR) was 26.7% in the hCG group and 31.3% in the control group.

Some authors evaluated the effect of hCG on the endometrium of cycles with FET and artificial cycles. Among them, Ben-Meir *et al*. (2010) performed a prospective randomized study of patients with FET in artificial cycles, with the objective of evaluating the positive or negative role of the use of recombinant hCG 250 mcg in 3 doses, from the day of initiation of PRG, transfer day, and 6 days after transfer. The clinical pregnancy rate (CPR) in group A was 28.2% *versus* 32.2% in group B; implantation rates were 12.7% and 14.9%, respectively. They concluded that there was no benefit in administering hCG in the luteal phase. Eftekhar *et al*. (2012) also performed a prospective randomized study; they administered IMP to patients in the case group and added 5000 IU of hCG on the day of PRG initiation and 5000 IU on the day of transfer. They did not find benefits of this regimen in the luteal phase.

In an observational study enrolling patients with a history of implantation failure and thin endometria, Davar *et al*. (2016) administered hCG daily to 28 patients starting on the 8^th^ day of the cycle in FET, and observed endometrial growth of 5.07 to 7.85 mm with 150 IU of intramuscular hCG starting on the 8^th^ day of the cycle in the proliferative phase until the day of transfer. Five patients in the case group achieved clinical pregnancies. The control group consisted of patients with previous failures and none achieved pregnancy.

Shiotani *et al.* (2017) performed a prospective randomized study with 173 patients divided into 2 groups of FET. All received transdermal E2 and vaginal progesterone (VP), and the embryos were transferred between the 17^th^ and the 20^th^ day of hormone therapy. In group A, 3000 IU of hCG were administered on the 17^th^, 20^th^, and 23^rd^ days of hormone therapy. In group B, no hCG was administered. The CPR and implantation rates were 37.5% and 25.3% in group A and *versus* 35.6% and 21.7%, in group B; the difference was not statistically significant.

The retrospective study conducted in China by Deng *et al*. (2020) analyzed the effect of intramuscular administration of hCG before the use of IMP in artificial cycles for FET (study group with 337 transfers and 364 in the control group, which were not given hCG). They observed a significant difference in live births rates (LBR) in favor of the group that used hCG (hCG group 49.9%; control group 39.6%, *p*=0.006).

Due to the possible direct and probably beneficial effect of hCG on the endometrium, we decided to administer choriogonadotropin alfa (Ovidrel^®^) in low daily doses (20 mcg/day) associated with VP or IMP in the luteal phase from the day of initiation of PRG for 12 days and assess the chances of pregnancy achieved with this protocol.

### Objectives

Primary: To evaluate the effect of low doses of recombinant hCG in the luteal phase of artificial cycles for FET, on the chances of pregnancy in patients ≤ 38 years old, with transfer of 2 top embryos 1 top embryo transferred (day 3, day 4 and 5-6 days).

Secondary: To evaluate the chances of pregnancy in the FET groups of artificial cycles using VP *versus* IMP.

## MATERIALS AND METHODS

All patients signed an informed consent form, authorizing the retrospective and anonymous use of their data.

The study was approved by the Ethics Committee of the Maternity Hospital Dona Iris, Goiânia, Goiás, Brazil, and was given certificate no. 50873921.2.0000.8058.

### Inclusion criteria

Patients who underwent FET without genetic tests in the period from January 2017 to October 2020 performed by the author, meeting the following criteria:

Age ≤ 38 years.

Endometrial thickness ≥ 7 mm

Indication of IVF/ICSI

Transfers of 2 or 1 top embryo in day 3 with (≥8 blastomeres with fragmentation less than 20% or absent), top morula in day 4 with (≥ 80% compaction) and blastocysts ≥3 BB, in 5-6 days (Gardner *et al*., 2002).

### Exclusion criteria

Exclusion stages:

1^st^ phase: Patients with the following endometrial preparation scheme in the proliferative phase were excluded: natural cycle, modified natural cycle, and stimulated cycles.

2^nd^ phase: cycles that did not transfer top embryos.

### Final material studied

Initially, we selected 192 cycles of FET featuring patients aged ≤ 38 years from January 2017 to October 2020 from the author’s practice. These patients accounted for approximately 13% of the FETs performed in a private clinic in the same age group in that period.

Phase 1 of exclusion: forty-nine were excluded for use of natural, modified natural, or stimulated cycles.

**Figure 1:**
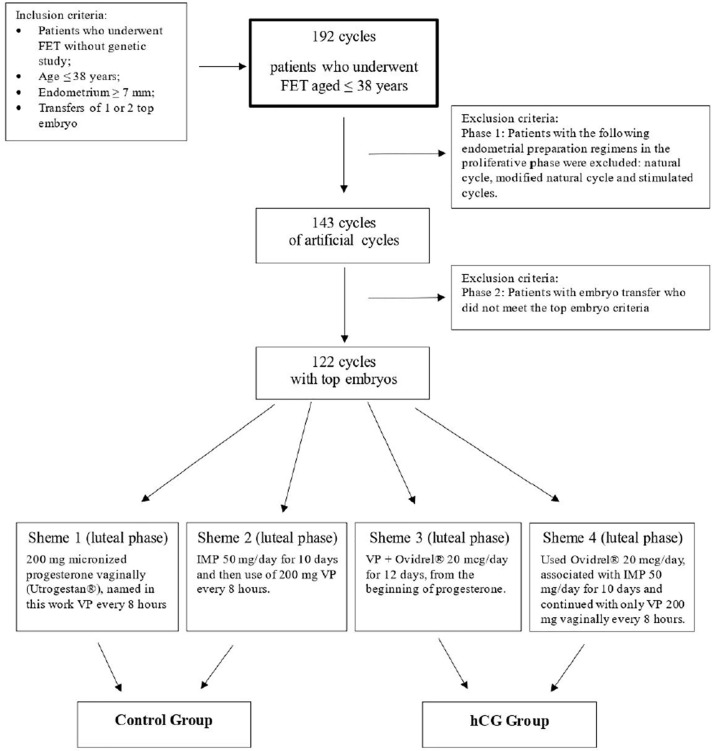
Patient enrollment flowchart.

Phase 2 exclusion: Of the remaining 143 cycles, 21 were excluded for featuring transfers without top embryos, leaving 122 patients who were the object of this study (Figure 1).

### Routine of freezing and thawing of embryos during the study period

The embryos were frozen using the vitrification technique, closed-system vitrification (Irvine®) method, and thawed using the same method.

### Routine for embryo transfer

Embryos that were frozen on day 3 or 4 were thawed in the morning one day before the transfer.

Embryos frozen on days 5 or 6 were thawed on the same day in the morning and transferred 3 to 7 hours after thawing.

The transfers were performed with a Guardia AccessET catheter from Cook medical approximately 30 minutes after the administration of oral diazepam 10 mg.

Soon after transfer the patients taken to a resting area, where they remained for approximately 10-15 minutes.

### Tests ordered after transfer

All patients underwent measurement of E2, PRG, and beta hCG levels on the day of initiation of PRG +11 (DP11) and +18 (DP18), which correspond approximately to 6 days and 13 days after blastocyst transfer, based on day 0, the day of transfer, according to the author’s routine.

### Protocol for initial endometrial preparation and maintenance

A - E2 valerate (E2 valerate, Primogyna^®^ 2 mg orally every 8 hours and rarely Estradot^®^ 100, 1 patch daily or Oestrogel^®^ 2 pumps every 6 hours, associated with E2 valerate).

### Luteal phase regimens

The protocol used during the study period was as follows:

Scheme 1: 200 mg micronized VP (Utrogestan^®^), every 8 hours.

Scheme 2: IMP 50 mg/day for 10 days and then, use of 200 mg VP every 8 hours.

Scheme 3: VP + Ovidrel^®^ 20 mcg/day for 12 days, from the beginning of PRG.

Scheme 4: Ovidrel^®^ 20 mcg/day, associated with IMP 50 mg/day for 10 days and continued with only VP 200 mg vaginally every 8 hours.

Two study groups were formed. One was the control group (CG), in which patients were not given hCG, with individuals on schemes 1 and 2; and a case group, named the hCG group, in which patients were given hCG, including individuals on schemes 3 and 4.

The chances of Beta hCG+ (beta ≥ 40 mIU/ml at 13-14 days post-transfer), CPR (presence of a gestational sac at 5-6 weeks of gestation), OPR (≥ 11 weeks of gestation), and LBR (≥22 weeks of gestation) were calculated for each group.

### Statistics

Continuous variables were expressed as mean± SD and analyzed with the t-test.

The chi-square test and Fisher’s exact test were used to compare categorical variables. Logistic regression analysis was conducted to evaluate the covariates affecting pregnancy rates.

Statistical package Graph Pad Prism 9.0 was used in the study.

## RESULTS

The chances of pregnancy by embryo transfer, Beta hCG+, CPR, OPR and LBR, per scheme of luteal phase preparation of artificial cycles for FET (groups 1, 2, 3, 4) are described in Table 1. The chances of LBR were 35.55%, 26.66%, 31.57% and 33.33%, respectively, in these groups. We also included the chance of multiple twin pregnancy. No significant difference was observed between groups (Table 1).

**Table 1:** Chances of pregnancy in FET of artificial cycles using the luteal phase scheme (1, 2, 3, 4).

	1 (n=45)	2 (n=15)	3 (n=38)	4 (n=24)	Anova (*p*)
**Beta hCG +**	23 (51.11%)	6 (40%)	20 (52.63%)	11 (45.83%)	0.3525
**Clinical Pregnancy**	22 (48.88%)	5 (33.33%)	17 (44.73%)	11 (45.83%)	0.3463
**Ongoing Pregnancy**	16 (35.55%)	4 (26.66%)	12 (31.57%)	8/24 (33.33%)	0.3456
**Live Birth Rate**	16 (35.55%)	4 (26.66%)	12 (31.57%)	8/24 (33.33%)	0.3456
**Multiple Pregnancy**	6/16 (37.50%)	2/4 (50%)	3/12 (25%)	2/8 (25%)	0.3365

1=vaginal progesterone (VP)

2=intramuscular progesterone (IMP)

3= VP + hCG

4=IMP + hCG

The analysis of the two luteal phase groups (CG and hCG group) revealed no significant difference in previous cycles with implantation failures, number of top embryos transfers, number of embryos transferred, average levels of E2 and PRG on the day of transfer +6 (DT6), percentage of transferences corresponding to day 3, day 4, days 5 and 6, average endometrial thickness before the start of PRG, percentage of transfers with 1 or 2 top embryos, and LBR in transfers with 1 or 2 top embryos. However, there was a significant difference in mean age in the two groups, with controls at 32.53 years and the hCG group at 30.91 years (*p*=0.0114) (Table 2).

**Table 2:** Variables that may affect the results in the two luteal phase groups (control group and hCG group).

Variables	CG n=60	hCG n=62	OR (95%IC)	Q
Age^*^	32.53±3,20	30.91±3.69		0.0114
**Cycles with prior implantation failure**	8 (13.33%)	6 (9.67%)	1.436 (0.439-4.567)	0.5799
E2 DT +6^*^ (pg/ml)	267.84±169.12	300.09±213.23		0.5206
Progesterone DT +6^*^ (ng/ml)	16.5±10	12.3±8.49		0.7529
b-hCG^*^ +6 (mIU/ml)	35.4±84.2	48.5±54.2		0.8954
**Day-3 embryo transfer (%)**	2 (3.33%)	01 (1.61%)	2.103 (0.238-30.92)	0.6157
**Day-4 embryo transfer (%)**	13 (21.66%)	12 (19.35%)	1.152 (0.4951-2.660)	0.8243
**Embryo transfer on days 5-6 (%)**	47 (78.33%)	49 (79.03%)	0.9592 (0.4077-2.268)	>0.9999
Embryos transferred^*^	1.95±0,64	2.16±0.54		0.0542
Endometrium (mm)^*^	9.38±1.97	9.16±1.53		0.5206
**Transfer of 2 top embryos (%)**	25 (41.66%)	25 (40.32%)	1.057 (0.4988-2.245)	>0.9999
**Transfer of 1 top embryo (%)**	35 (58.33%)	37 (59.67%)	0.9459 (0.4454-2.005)	>0.9999

OR=odds ratio; CG=control group

* mean and standard deviation.

E2= estradiol; DT+6= 6 days after embryo transfer.

We did not observe significant differences in the rates of positive beta hCG tests, CPR, LBR and percentage of multiple pregnancies in the groups in terms of odds ratios (OR), confidence interval (CI), and Fisher’s test results. The LBR in the control group (33.33%) was similar to the one found in the hCG group (32.25%) (Table 3).

**Table 3:** Chances of pregnancy by study group (control group versus hCG group) and percentage of multiple pregnancies in the groups.

	CG (n=60)	hCG group (n=62)	OR (95%IC)	Q
Beta hCG+	29 (48.33%)	31 (50%)	0.9355 (0.4517-1.930)	0.8586
Clinic Pregnancy	27 (45%)	28 (45.16%)	0.9935 (0.4769-2.067)	>0.9999
Ongoing Pregnancy	20 (33.33%)	20 (32.25%)	1.050 (0.4949-2.230)	>0.9999
Live Birth Rate	20 (33.33%)	20 (32.25%)	1.050 (0.4949-2.230)	>0.9999
Multiple Pregnancy#	08/20 (40%)	05/20 (25%)	2.000 (0.5037-7.590)	0.5006

OR=odds ratio, CG=control group.

#All multiple pregnancies were twins (twin pregnancy)

The chances of pregnancy in the groups given VP and IMP were not significantly different. The LBR was 33.73% in the VP group and 30.76% in the IMP group (Table 4).

**Table 4:** Chances of pregnancy by study group, vaginal progesterone (VP) versus intramuscular progesterone (IMP), with or without a prescription of hCG.

	VP (83)	IMP (39)	OR	CI	*p*
Beta hCG+	43 (51.80%)	17 (43.58%)	1.38526	0.64275-3.02472	0.40622
Clinic Pregnancy	39 (46.98%)	16 (41.02%)	1.26915	0.58704-2.78953	0.54597
Ongoing Pregnancy	28 (33.73%)	12 (30.76%)	1.13933	0.50576-2.66216	0.75596
Live Births Rate	28 (33.73%)	12 (30.76%)	1.13933	0.50576-2.66216	0.75596
Multiple Pregnancy	09/28 (32.14%)	04/12 (33.33%)	1.04384	0.30977-4.21641	0.94714

VP=vaginal progesterone, IMP=intramuscular progesterone.

**Figure 2:**
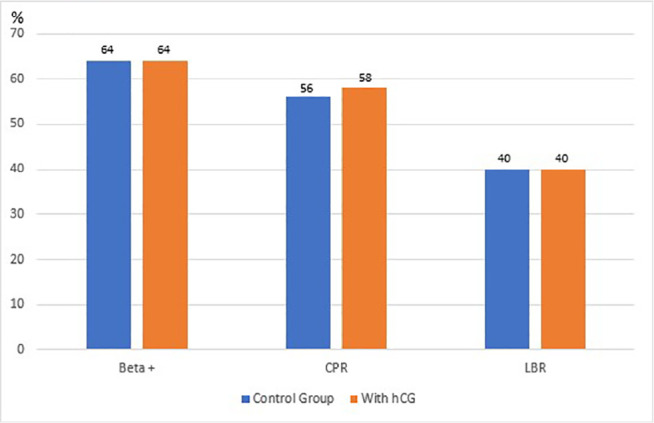
Pregnancy rates with 2-top-embryo transfers (days 4-6) with or without hCG use. CPR=clinical pregnancy rate LBR=live birth rate.

The chances of having a positive beta hCG test from a transfer with top embryos from 4-6 days, CPR, and LBR of patients not prescribed and individuals given hCG were 64%/64%, 56%/58%, and 40%/40%, respectively, with no significant difference in pregnancies (Figure 2).

## DISCUSSION

Several authors (Fishel *et al*., 1984; Woodward *et al.*, 1993) demonstrated the ability of the fresh blastocyst in the implantation phase to produce hCG, and this production was time dependent in relation to the fertilization process, as demonstrated by Woodward *et al.* (1993). Other authors have demonstrated the presence of receptors for this hormone in the endometrium (Reshef *et al*., 1990).

The effect of hCG produced by fresh blastocysts during implantation on the endometrium was well documented by Licht *et al*. (2001), with the hormone showing positive effects on implantation, in terms of vascularization and production of proteins, kinins and others, in addition to demonstrating a feedback effect on the process of trophoblastic invasion. These effects acted directly or indirectly (through stimulatory action on the corpus luteum, increasing the production of E2 and PRG, hormones with direct action upon the endometrium) upon the endometrium. Perrier d’Hauterive *et al*. (2007) wrote about the interaction between blastocyst hCG and endometrial LH/hCG receptors, showing its role in the implantation process.

The use of hCG in the luteal phase of FET in artificial cycles was suggested by some authors (Ben-Meir *et al*., 2010) due to the likely positive endometrial effect on the implantation process. These authors prescribed 250 mcg of recombinant hCG on the day of PRG initiation, 6 days after transfer, and did not observe differences in the chances of pregnancy. Other authors, with or without recombinant hCG, with different doses and different time intervals, also failed to find a positive effect on CPR or LBR (Eftekhar *et al*., 2012; Shiotani *et al*., 2017; Lee *et al*., 2017). Du *et al*. (2020) retrospectively studied the CPR and the LBR in two groups of patients undergoing IVF/ICSI and subsequent FET with artificial cycles with a previous diagnosis of endometriosis. They compared patients given hCG (355 cycles) to controls (296 cycles) and reported higher CPR in the hCG group (57.7%/49%, *p*=0.027). However, the LBR was not significantly different (45.6%/38.5%, *p*=0.08).

The use of daily low doses of recombinant hCG associated with VP or IMP might show different results when compared to studies performed previously, in which higher doses were administered with intervals between injections. We did not find any improvements in pregnancy rates by adding hCG in the luteal phase. The CPR and LBR of patients not prescribed and in individuals prescribed hCG in the luteal phase were approximately 45% and 33%, respectively, similar to what Eftekhar *et al*. (2012), Lee *et al*. (2017), and Shiotani *et al*. (2017) reported. Higher rates might be explained by the fact that they only used embryos of excellent morphological quality in their studies (Du *et al*., 2020).

An additional finding of our study was the fact that IMP had no benefit over VP (Table 4). Our findings resonate with the conclusions reported by Hershko Klement *et al*. (2018), Abdelhakim *et al*. (2020), and Polat *et al*. (2020) with the use of similar doses, and differ from the data published by Devine *et al*. (2021), who found IMP at a dose of 50 mg/day outperformed VP at a dose of 400 mg/day. In our study, the dose of VP was 600 mg/day, which may explain the difference vis-à-vis the results described by Devine *et al*. (2021).

The analysis of the variables that might affect the rates in each group did not reveal significant differences, with the exception of mean age. The lack of a significant difference in hormone levels (E2, PRG, beta hCG) may be explained by the fact that they only reflected the averages between pregnant and nonpregnant women and that the cycles were artificial. In artificial cycles, due to the absence of a corpus luteum, the tendency is for the mean values of E2 and PRG on day 6 to be similar, with or without the use of hCG. The prognostic quality of these hormones (E2, PRG) is known for CPR and LBR in the presence of a corpus luteum in fresh embryo transfers, when blood is collected approximately 6 days after day 5 blastocysts (Florêncio *et al*., 2018).

So, our attempts to achieve higher LBR with the use of daily low doses of hCG in the luteal phase of artificial cycles of FET proved unsuccessful with this scheme. Other schemes, such as administration of hCG before the start of PRG in artificial cycles of FET, will probably evaluated in the future to try and improve endometrial conditions, as suggested by Deng *et al*. (2020).

## CONCLUSIONS

In this FET study, there was no significant difference between patients prescribed and individuals not prescribed daily low-dose hCG in the luteal phase. There was also no significant difference in the groups that received IMP or VP.
